# Modeling the Effect of Modified Atmospheres on Conidial Germination of Fungi from Dairy Foods

**DOI:** 10.3389/fmicb.2017.02109

**Published:** 2017-10-31

**Authors:** Nicolas Nguyen Van Long, Valérie Vasseur, Olivier Couvert, Louis Coroller, Marion Burlot, Karim Rigalma, Jérôme Mounier

**Affiliations:** ^1^Laboratoire Universitaire de Biodiversité et Ecologie Microbienne, IBSAM, ESIAB, Université de Brest, EA 3882, Plouzané, France; ^2^Laboratoire Universitaire de Biodiversité et Ecologie Microbienne, IBSAM, UMT Spore Risk, ESIAB, Université de Brest, EA 3882, Quimper, France

**Keywords:** predictive mycology, carbon dioxide increase, dioxygen reduction, modified atmosphere, dairy foods

## Abstract

Modified atmosphere packaging (MAP) is commonly applied to extend food shelf-life. Despite growth of a wide variety of fungal contaminants has been previously studied in relation to modified-atmospheres, few studies aimed at quantifying the effects of dioxygen (O_2_) and carbon dioxide (CO_2_) partial pressures on conidial germination in solid agar medium. In the present study, an original culture method was developed, allowing microscopic monitoring of conidial germination under modified-atmospheres in static conditions. An asymmetric model was utilized to describe germination kinetics of *Paecilomyces niveus, Mucor lanceolatus, Penicillium brevicompactum, Penicillium expansum*, and *Penicillium roquefoti*, using two main parameters, i.e., median germination time (τ) and maximum germination percentage (*P*_*max*_). These two parameters were subsequently modeled as a function of O_2_ partial pressure ranging from 0 to 21% and CO_2_ partial pressure ranging from 0.03 to 70% (8 tested levels for both O_2_ and CO_2_). Modified atmospheres with residual O_2_ or CO_2_ partial pressures below 1% and up to 70%, respectively, were not sufficient to totally inhibit conidial germination,. However, O_2_ levels < 1% or CO_2_ levels > 20% significantly increased τ and/or reduced *P*_*max*_, depending on the fungal species. Overall, the present method and results are of interest for predictive mycology applied to fungal spoilage of MAP food products.

## Introduction

Food spoilage by fungi leads to important economic losses and can raise safety issues in the case where fungal spoilers are mycotoxin-producers (Legan, 1993; Garcia et al., 2009). Asexual spores (conidia) are mainly responsible for accidental contaminations of food products as they are produced at high numbers and easily dispersed by airflows (Rosas et al., 1993; Wyatt et al., 2013). As the conidial germination precedes growth on food, thus delaying or preventing this developmental stage can help to extend food shelf-life. On the other hand, fungal development in foods can be controlled by different abiotic factors (also called hurdle technologies) which can be combined or not. These abiotic factors include food intrinsic factors, mainly water activity (*a*_*w*_), pH, redox potential, available nutrients and antimicrobial substances, and extrinsic factors, mainly temperature and gaseous composition (Huis in't Veld, 1996). Modification of the gaseous composition in food packaging headspace, with the aim to extend its shelf life, is a widespread practice which encompasses controlled-atmosphere storage (CAS) and modified-atmosphere packaging (MAP) (Caleb et al., 2012). CAS is generally used for large batches of fruits or vegetables stored in rooms where the gas mixture is continuously controlled and renewed (Littlefield et al., 1966; Yackel et al., 1971). In contrast, MAP involves an initial modification of the headspace composition within the packaging of individual products without any further control during storage (Ooraikul, 2003). In both methods, atmosphere modifications consist mainly in reduction of dioxygen (O_2_) or increase of carbon dioxide (CO_2_) partial pressures as compared to atmospheric air composition. These modifications contribute to reduce the physico-chemical and microbiological deteriorations of the packaged food (Chaix et al., 2015).

Predictive modeling (predictive mycology) can be applied to quantify the influence of hurdle technologies on fungal development and mycotoxin production in food products (Leistner and Gorris, 1995; Dantigny et al., 2005a). In predictive mycology, the use of Gamma-type models, assuming multiplicative effects of the different factors, provide cardinal values which are relevant for food safety management systems (Dagnas and Membré, 2013). To date, cardinal models were successfully used to describe effects of temperature, water activity and pH on conidial germination and radial growth (Dagnas and Membré, 2013). Since modified-atmospheres are commonly used as a food preservation technology (McMillin, 2008), measuring the respective effects of O_2_ and CO_2_ is required to apply the Gamma concept (Zwietering et al., 1993) for CAS and MAP foods.

In this perspective, the present study attempted to provide an experimental set up which allows conidial germination monitoring on solid medium kept under modified-atmospheres as well as mathematical models describing O_2_ and CO_2_ effects on the conidial germination kinetics of five fungal species which are encountered in dairy foods, namely *Paecilomyces niveus, Mucor lanceolatus, Penicillium brevicompactum, Penicillium expansum*, and *Penicillium roqueforti*.

## Materials and methods

### Culture medium

The culture medium used throughout the experiments was PDA supplemented with 10.13% (w/w) glycerol to reach 0.980 *a*_*w*_. Citric acid monohydrate (0.1 M) and sodium phosphate dibasic (0.2 M) solutions (Sigma-Aldrich, Saint-Louis, MO, USA) (3/2, v/v) were also used to buffer the culture medium at pH 4.2. Agar medium pH was measured after autoclaving and solidification of three samples of each culture medium batch, using a pH surface-electrode (SF 113, VWR, Radnor, PA, USA) with an accuracy of 0.01 pH unit. The *a*_*w*_ was also verified on three samples of each culture medium batch at 20°C using a Tunable Diode Laser *a*_*w*_-meter (TDL Aqualab, Decagon Devices, Pullman, WA, USA) calibrated using sodium chloride (Sigma-Aldrich, Saint-Louis, MO, USA) solutions of known *a*_*w*_ provided by the manufacturer with an accuracy of 0.005 *a*_*w*_ unit.

### Fungal strains and conidial production

Fungal strains used in the present study were obtained from the Université de Bretagne Occidentale Culture Collection (UBOCC, Plouzané, France). *P. niveus* UBOCC-A-11024 was isolated from cow milk, *M. lanceolatus* UBOCC-A-109153 was isolated from cheese while *P. brevicompactum* UBOCC-A-110007, *P. expansum* UBOCC-A-110032 and *P. roqueforti* UBOCC-A-113022 were isolated from soft, fresh and Roquefort cheeses, respectively. Stock cultures were routinely cultured on potato dextrose agar (PDA, Difco, Becton Dickinson, Sparks, MD, USA) at 25°C. Conidia suspensions of the five fungal species were obtained as previously described (Nguyen Van Long et al., 2017). Briefly, conidia were harvested from cultures incubated for 10 days at 25°C on PDA medium at 0.980 *a*_*w*_ and pH 4.2. Conidial concentrations were determined using a haemocytometer (Malassez, Preciss, Paris, France) and standardized at 1.10^5^ conidia/mL in glycerol (Thermo Fischer Scientific, Waltham, MA, USA) solutions adjusted to 0.980 *a*_*w*_ and pH 4.2 prior to further use.

### Gas mixtures

Gas mixtures were prepared by dilution of pressurized air into diving cylinders (15L-cylinder twin valve, Aqualung, Carros, France). For O_2_ adjustment, pressurized air was diluted with N_2_ (Alphagaz2 Azote, Air Liquide S.A, Paris, France) in order to obtain eight different mixtures with O_2_ partial pressures ranging from 0 to 21% (Table 1). A 0%-O_2_ partial pressure was obtained using O_2_ absorber sachets (ATCO V30002, Laboratoires Standa, Caen, France) while a 21%-O_2_ partial pressure was obtained with air. For CO_2_ adjustment, pressurized air was diluted with CO_2_ (Alphagaz 1 dioxyde de carbone, Air Liquide S.A, Paris, France) in order to obtain eight different mixtures with CO_2_ partial pressure ranging from atmospheric partial pressure (i.e., 0.04%) to 70%, with O_2_ partial pressure kept at 5% by completing the mixtures with N_2_ (Table 1). After preparation, the gas composition of the different mixtures was controlled using a headspace gas analyzer equipped with a Zirconial/dual beam infrared sensor (Checkmate 3, Dansensor, Ringsted, Denmark) with an accuracy of 0.01 and 0.8% for O_2_ and CO_2_ measurements, respectively.

**Table 1:** Mean values ± standard deviation of dioxygen (O_2_) or carbon dioxyde (CO_2_) partial pressures (%) measured in three replicates of modified-atmosphere culture devices containing PDA plates inoculated with *Paecilomyces niveus* UBOCC-A-11024, *Mucor lanceolatus* UBOCC-A-109153, *Penicillium brevicompactum* UBOCC-A-110007, *Penicillium expansum* UBOCC-A-110032 or *Penicillium roqueforti* UBOCC-A-113022, depending on targeted partial pressures (at 25°C) and maximum tested duration of experiments.

**Gas tested**	**Targeted partial pressure (%)**	**Mean ± standard deviation of measured partial pressure (%)**	**Maximum duration tested (h)**
O_2_	0.00	0.00 ± 0.00^*^	>720
	0.25	0.36 ± 0.05	66.33
	0.50	0.61 ± 0.06	58.00
	0.75	0.87 ± 0.06	43.33
	1.00	1.18 ± 0.25	75.50
	5.00^**^	5.10 ± 0.34	31.17
	10.00	10.34 ± 0.46	57.83
	21.00	20.32 ± 0.15	31.22
CO_2_	0.00^**^	0.01 ± 0.03	31.17
	10.00	10.31 ± 1.37	42.05
	20.00	22.34 ± 1.25	69.75
	30.00	28.39 ± 1.22	65.32
	40.00	39.55 ± 1.77	82.62
	50.00	49.25 ± 1.97	172.17
	60.00	58.48 ± 2.04	47.67
	70.00	69.00 ± 1.76	148.50

*When O_2_ was the studied factor, CO_2_ partial pressure was below 0.04%. When CO_2_ was the studied factor, O_2_ partial pressure was at 5%*.

(*) * O_2_ absorber included*.

(**) *Both conditions (5.00% O_2_ or 0.00% CO_2_) correspond to the same experiment*.

### Conidial germination assessment under modified-atmospheres

#### Modified-atmosphere culture device

**Figure 1:**
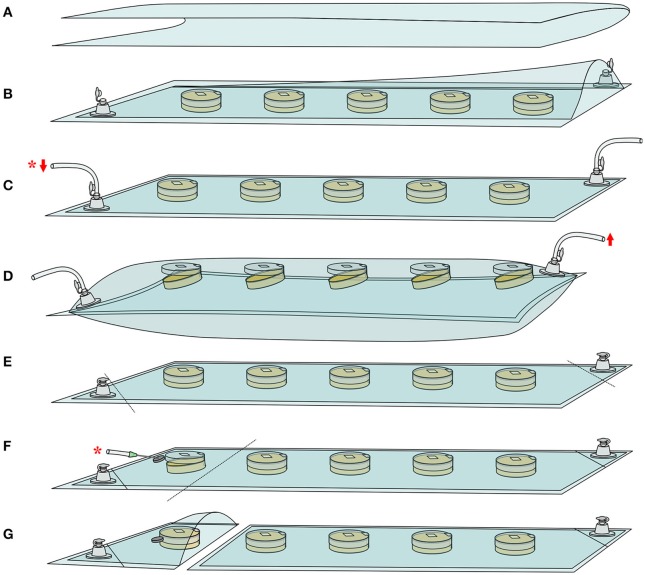
Experimental device developed to monitor conidial germination on solid agar medium under modified atmosphere. See Material and Methods' section for details. **(A)** Lengthwise folding of high oxygen barrier film. **(B)** Thermosealing, fixation of non-return PVC valves and placement of inoculated plates. Successive steps of **(C)** vacuum (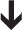) and **(D)** filling (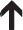) with the gas mixture. Vacuum steps are performed with a downstream gaseous composition measurement (^*^). **(E)** Isolation of the non-return valves by thermosealing prior to incubation. **(F)** Isolation of a dish and gaseous composition measurement (^*^). **(G)** Extraction of the isolated dish for germination assessment.

To evaluate effects of O_2_ and CO_2_ partial pressures on conidial germination, a simple device was developed. This device was designed to allow germination assessment in solid culture medium under modified-atmosphere injected at the start of the experiment. Moreover, this device allowed to maintain a constant gas composition within the incubation time and the gaseous composition was controlled each time spore germination was assessed. It consisted of a rectangular plastic bag made from bioriented polyester film (Biaxer 55XX, Wipak, Bomlitz, Germany) with high oxygen barrier (oxygen permeability <3 cm^3^.m^−2^.d^−1^.bar^−1^ measured at 23°C and 50% relative humidity) equipped with two PVC non-return valves (03-325, Carmo A.S, Espergærde, Denmark), as described in Figure 1. To prepare plastic bags, a 420 × 600-mm rectangle of plastic film was first folded in half lengthwise (Figure 1A) prior to sealing of two adjacent sides using a manual thermosealer (BS600, Tecnimodern automation, Saint-Germain-Laval, France). Secondly, two circular holes (5 mm diameter) were punched into opposite corners of the bag. A valve was then fixed on each hole with PVC glue (Carmo Seal, Carmo A.S, Espergærde, Denmark) (Figure 1B).

#### Inoculation and setting of gas composition

For each studied fungal species and gas mixture, a batch of five 55-mm diameter Petri dishes (Gosselin, Hazebrouck, France) was inoculated in three independent replicates. For each condition, a second batch was also inoculated in three independent replicates 10 h following inoculation of the first batch. The use of a second batch allowed the monitoring of conidial germination at any incubation time, including periods outside working hours. Conidia were surface-plated on 10 mL of PDA with 36 μL of a 1.10^5^ conidia/mL suspension in order to obtain ~1 conidium/mm^2^ of culture medium. Immediately after inoculation, Petri dishes were aligned in the bag prior to sticking the Petri dish top lid to the bag inner side with double-sided tape (10 × 10 mm). The bag was sealed and connected to a vacuum pump (to accelerate the complete filling of the bags) and to the gas mixture cylinder through the respective valves (Figure 1C). Expelled gas composition was continuously measured downstream of the vacuum pump. Using this set-up, three successive steps of vacuum treatment (Figure 1C) and filling with the desired gas mixture (Figure 1D) were performed, i.e., until injected and expelled gas had similar composition. During gas filling steps, bags were allowed to swell until the top lid (taped to the inner side of the bag) opened, enabling Petri dishes headspaces to be flushed with the injected gas (Figure 1D). At the end of gas filling steps, bags were deflated to atmospheric pressure (final bag volume of 1.19 L) and valves isolated from the system using thermosealing (Figure 1E). Finally, bags were incubated at 25°C (KB 240, Binder GmbH, Tuttlingen, Germany).

#### Conidial germination assessment

Prior to conidial germination assessment, one Petri dish was isolated from the other by thermosealing (Figure 1F). The gaseous composition of the isolated bag section was measured by inserting the gas analyzer needle through a foam septum (Figure 1F). After removing the plate from the bag, the remaining packed Petri dishes were immediately put back into the incubator for further assessments (Figure 1G). Conidia germination was examined directly onto the agar plate using phase-contrast microscopy (BX51 microscope, Olympus, Tokyo, Japan) as previously described (Nguyen Van Long et al., 2017). A minimum of 100 conidia was counted for each agar plate to determine the percentage of germinated conidia as a function of time. Conidia were considered germinated when germ tube length was greater or equal to that of swollen conidia (Dantigny et al., 2006). Conidial germination assessment began after an incubation time ranging from 7.5 to 172 h and was performed at time intervals depending on the fungal species and gaseous composition.

### Data modeling and statistical analysis

#### Conidial germination kinetic modeling

Kinetic of germinated conidia percentage *P*_(*t*)_ (%) as a function of incubation time *t* (h) was modeled using a primary asymmetric model Equation (1) (Dantigny et al., 2011):

(1) $$\begin{array}{l} P_{\left(t\right)}\;=\;P_{\max}\cdot \left(1-\frac{1}{1+{\left(\frac{t}{\tau }\right)}^{d}}\right) \end{array}$$

where *P*_*max*_ (%) is the maximum percentage of germinated conidia. The median germination time τ (h) is the time when *P* equals half of *P*_*max*_. The parameter *d* (without unity) is a shape parameter. The model was fitted by minimizing the sum of squares of the residuals (*lsqcurvefit function*, Matlab 2014 The Mathworks Inc., USA). The 95% confidence intervals were calculated using *nlparci function* from Matlab Statistic Toolbox (Matlab R2016, The Mathworks, Natick, USA). The determination coefficient (r^2^) and the root mean square error (RMSE) were calculated to evaluate fitting performances.

#### Modeling the effect of O_2_ partial pressure on germination kinetic parameters

The median germination time τ (h) was modeled as a function of O_2_ partial pressure [O_2_] (%) with Equation (2):

(2) $$\begin{array}{l} \tau =\;\tau_{atm}\cdot \left(1+\frac{{\left[O_{2}\right]}_{50}}{\left[O_{2}\right]}\right) \end{array}$$

where τ_*atm*_ (h) is the value of τ under O_2_ atmospheric partial pressure (i.e., 20.9 %) and [*O*_2_]_50_ (%) is the O_2_ partial pressure at which τ is twice as long as τ_*atm*_. In addition, *P*_*max*_ (%) was modeled as a function of [O_2_] (%) with Equation (3):

(3) $$\begin{array}{l} P_{\max}=\;P_{\max_{atm}}\cdot \frac{1}{\left(1\;+\;\frac{{\left[O_{2}\right]}_{50}}{\left[O_{2}\right]}\right)} \end{array}$$

where *P*_*maxatm*_ (%) is the *P*_*max*_ value under atmospheric partial pressure of O_2_ and [*O*_2_]_50_ (%) is the O_2_ partial pressure at which *P*_*max*_ is half of *P*_*maxatm*_. The models were fitted by minimizing the sum of squares of the residuals as described in the previous section.

#### Modeling the effect of CO_2_ partial pressure on germination kinetic parameters

The reciprocal of the median germination time τ^−1^ (h^−1^) was modeled as a function of CO_2_ partial pressure [CO_2_] (%) with a model based on a reparameterized Monod-type Equation (4) (Dantigny et al., 2005b):

(4) $$\begin{array}{l} \tau^{-1}\;=\;\tau_{atm}^{-1}\\ \;\cdot \left(\frac{{\left[CO_{2}\right]}_{50}\cdot \left({\left[CO_{2}\right]}_{MI}-\left[CO_{2}\right]\right)}{{\left[CO_{2}\right]}_{50}\cdot {\left[CO_{2}\right]}_{MI}-\;2\cdot {\left[CO_{2}\right]}_{50}\cdot \left[CO_{2}\right]\;+\;{\left[CO_{2}\right]}_{MI}\cdot \left[CO_{2}\right]}\right) \end{array}$$

where $\tau_{atm}^{-1}$ (h^−1^) is the value of τ^−1^ under atmospheric [CO_2_], [*CO*_2_]_50_ (%) is the CO_2_ partial pressure at which τ^−1^ is half of $\tau_{atm}^{-1}$ and [*CO*_2_]_*MI*_ (%) is the CO_2_ partial pressure at which τ^−1^ is equal to zero, namely the minimal inhibitory CO_2_ partial pressure. In addition, the *P*_*max*_ parameter was modeled as a function of [CO_2_] (%) with Equation (4) where $\tau_{atm}^{-1}$ is substituted by *P*_*maxatm*_ (%) which is the value of *P*_*max*_ under atmospheric [CO_2_], [*CO*_2_]_50_ (%) is the CO_2_ partial pressure at which *P*_*max*_ is half of *P*_*maxatm*_ and [*CO*_2_]_*MI*_ (%) is the CO_2_ partial pressure at which *P*_*max*_ is equal to zero.

## Results

### Validation of the modified-atmosphere culture device

The modified-atmosphere culture device developed in the present work allowed simultaneous measurements of the gaseous composition in Petri dish headspace and conidial germination assessment on solid medium. Whatever the tested conditions, gas composition remained stable throughout all experiments as shown in Table 1. Concerning atmospheres with reduced O_2_ partial pressures, the targeted partial pressures could be maintained up to 75.5 h without oxygen absorber or up to 30 days using the ATCO V30002 oxygen absorber (Table 1). For O_2_ partial pressures below 1%, slight differences were observed between targeted and measured partial pressures. Concerning MAP with increased CO_2_ partial pressures, each targeted CO_2_ partial pressure was also accurately obtained and maintained for incubation times up to 172.2 h (Table 1).

### Modeling of conidial germination kinetics

**Figure 2:**
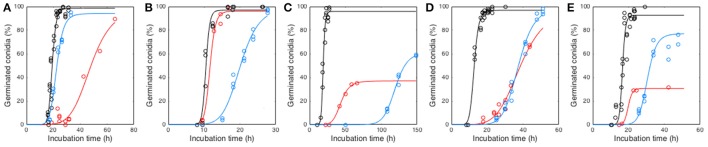
Conidial germination kinetics of *Paecilomyces niveus* UBOCC-A-11024 **(A)**, *Mucor lanceolatus* UBOCC-A-109153 **(B)**, *Penicillium brevicompactum* UBOCC-A-110007 **(C)**, *Penicillium expansum* UBOCC-A-110032 **(D)** and *Penicillium roqueforti* UBOCC-A-113022 **(E)** on PDA (pH 4.2, 0.980 *a*_*w*_) at 25°C under atmospheric air i.e., 21% O_2_ and 0.03% CO_2_ (black), 0.36% O_2_ and 0.03% CO_2_ (red) or 69% CO_2_ and 5% O_2_ (blue). Circles represent observed germination percentages (%) and solid lines are obtained from the Equation (1) model.

Conidia were able to germinate in all studied conditions within the incubation time (i.e., 30 days), except at 0% O_2_ (Supplementary Tables 1, 2). Hence, germination kinetics were obtained for seven different O_2_ partial pressures and seven different CO_2_ partial pressures for each tested species. As illustrated in Figure 2, conidial germination kinetics had a sigmoidal shape and was modeled by Equation (1). At a O_2_ level of 0%, *P*_*max*_ was considered to be 0% and τ longer than the incubation time while *d* was not estimated. Independently of the fungal species, germination was the fastest under atmospheric conditions whereas germination was the slowest at 0.36% O_2_ or 69.00% CO_2_ (Figure 2). Reduction of O_2_ or increase of CO_2_ below or above atmospheric pressure respectively increased τ values. They ranged between 18.22 and 47.88 h, 10.29 and 20.12 h, 18.61 and 130.69 h, 13.80 and 38.56 h and 16.78 and 30.53 h for *P. niveus, M. lanceolatus, P. brevicompactum, P. expansum*, and *P. roqueforti*, respectively. Moreover, O_2_ reduction or CO_2_ increase decreased *P*_*max*_ values as low as 37.05% for *P. brevicompactum* and 30.66% for *P. roqueforti*, whereas *P*_*max*_ of other species remained unaffected. The *d* parameter was estimated at 12.15 ± 3.09, 12.87 ± 3.61, 12.27 ± 3.61, 10.07 ± 3.44, 12.90 ± 4.78 for *P. niveus, M. lanceolatus, P. brevicompactum, P. expansum*, and *P. roqueforti*, respectively. As low variations of *d* were observed, this parameter was not modeled as a function of gaseous composition.

### Modeling the effects of oxygen and carbon dioxide on conidial germination

#### Modeling the effect of oxygen partial pressure on median germination time τ

**Figure 3:**
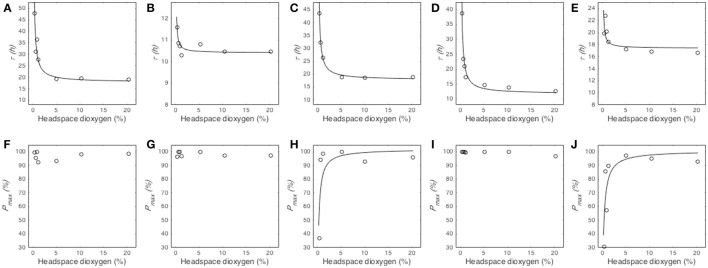
Influence of O_2_ partial pressure (%) in headspace on the median germination time τ (h) and maximum germination percentage *P*_*max*_ (%) of *Paecilomyces niveus* UBOCC-A-11024 **(A,F)**, *Mucor lanceolatus* UBOCC-A-109153 **(B,G)**, *Penicillium brevicompactum* UBOCC-A-110007 **(C,H)**, *Penicillium expansum* UBOCC-A-110032 **(D,I)** and *Penicillium roqueforti* UBOCC-A-113022 **(E,J)** on PDA medium (pH 4.2, 0.980 *a*_*w*_) at 25°C. Observed parameters (open circle), model fitted with Equation (2) **(A–E**, solid line) or Equation (3) **(F–J**, solid line).

The median germination time (τ) remained stable at O_2_ partial pressures above a certain threshold dependent of the studied fungal species while it dramatically increased at low O_2_ partial pressures (Figure 3). The model Equation (2) successfully described this effect for the five tested species and provided two parameters, namely the median germination time under atmospheric oxygen partial pressure (τ_*atm*_) and the O_2_ partial pressure at which τ is twice as long as τ_*atm*_ ([*O*_2_]_50_) (Table 2). Regarding τ_*atm*_, it varied between 10.41 and 17.87 h, depending on the fungal species. *M. lanceolatus* conidia (τ_*atm*_ = 10.41 ± 0.08 h) and *P. expansum* conidia (τ_*atm*_ = 11.70 ± 0.65 h) germinated more rapidly than the other tested species under atmospheric conditions. Indeed, *P. niveus, P. brevicompactum* and *P. roqueforti* conidia had median germination times of ~17 h, with 17.87 ± 0.86 h, 17.76 ± 0.29 h and 17.39 ± 0.56 h, respectively. Regarding [*O*_2_]_50_, an O_2_ partial pressure lower than 1% was necessary to double τ for all studied species. Indeed, the highest values of [*O*_2_]_50_ were estimated at O_2_ levels of 0.75 ± 0.08%, 0.60 ± 0.06%, and 0.52 ± 0.02% for *P. expansum, P. niveus* and *P. brevicompactum*, respectively. For *P. roqueforti* and *M*. lanceolatus, the estimated values of [*O*_2_]_50_ (0.07 ± 0.02% O_2_ and 0.03 ± 0.01% O_2_, respectively) were below the lowest partial pressures tested without using oxygen absorber.

**Table 2:** Secondary model parameters ± standard deviation obtained by fitting Equation (2) to median germination times τ (h) or Equation (3) to maximum germination percentages *P*_*max*_ (%) of *Paecilomyces niveus* UBOCC-A-11024, *Mucor lanceolatus* UBOCC-A-109153, *Penicillium brevicompactum* UBOCC-A-110007, *Penicillium expansum* UBOCC-A-110032 and *Penicillium roqueforti* UBOCC-A-113022 as a function of O_2_ partial pressure in headspace (%).

**Fungal species**	**Parameters describing the effect of O_2_ partial pressure on *τ***				**Parameters describing the effect of O_2_ partial pressure on *P_*max*_***			
	***τ_*atm*_ (h)***	**[*O_2_*]*_50_ (%)***	***r*^2^**	**RMSE**	***P_*maxatm*_ (%)***	**[*O_2_*]*_50_ (%)***	***r*^2^**	**RMSE**
*P. niveus*	17.87 ± 0.86	0.60 ± 0.06	0.95	2.45	–	–	–	–
*M. lanceolatus*	10.41 ± 0.08	0.03 ± 0.01	0.62	0.23	–	–	–	–
*P. brevicompactum*	17.76 ± 0.29	0.52 ± 0.02	0.99	0.79	98.1 ± 5.5	0.25 ± 0.10	0.54	14.0
*P. expansum*	11.70 ± 0.65	0.75 ± 0.08	0.95	1.92	–	–	–	–
*P. roqueforti*	17.39 ± 0.56	0.07 ± 0.02	0.45	1.68	99.2 ± 5.1	0.32 ± 0.09	0.68	13.0

*The parameters τ_atm_ (h) and P_maxatm_ are the values of τ or P_max_ respectively in atmospheric conditions and the parameter [O_2_]_50_ (%) is the O_2_ partial pressure at which τ is twice as long as τ_atm_ or P_max_ is half of P_maxatm_. The accuracy of the model was evaluated using the root mean square error (RMSE) and the determination coefficient r^2^. (–) Model not adjusted*.

#### Modeling the effect of oxygen partial pressure on maximum percentage of germination P_max_

The effect of O_2_ partial pressure on the maximum percentage of germination (*P*_*max*_) varied according to the tested fungal species (Figures 3F–J). For *P. niveus, M. lanceolatus* and *P. expansum, P*_*max*_ did not vary between 0.25 and 21% O_2_ partial pressure while it was prevented at 0% O_2_ level (Figures 3F,G,I). Given this binary effect, the effect of O_2_ on *P*_*max*_ was not described by Equation (3) for these species (Table 2). For *P. brevicompactum* and *P. roqueforti, P*_*max*_ remained unaffected at O_2_ partial pressures above 0.75 and 0.5% respectively, followed by an important decrease in *P*_*max*_ below these thresholds (Figures 3H,J). Our model Equation (3) successfully described this effect for both species(Table 2) and allowed us to determine the maximum germination percentage under atmospheric O_2_ partial pressure (*P*_*maxatm*_) and the O_2_ partial pressure at which *P*_*max*_ is half of *P*_*maxatm*_ ([*O*_2_]_50_). *P*_*maxatm*_ and [*O*_2_]_50_ were estimated to be 100% for both species and, 0.25 ± 0.10% and 0.32 ± 0.09% O_2_, for *P. brevicompactum* and *P. roqueforti*, respectively.

#### Modeling the effect of carbon dioxide partial pressure on reciprocal of median germination time τ^−1^

**Figure 4:**
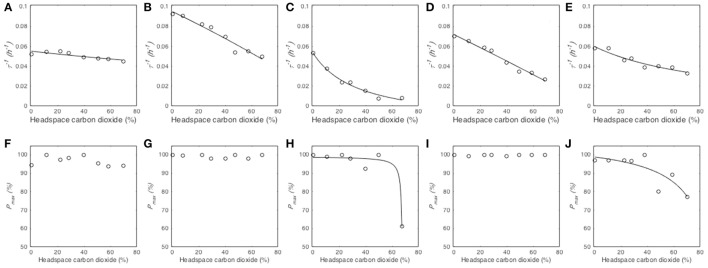
Influence of CO_2_ partial pressure (%) in headspace on the reciprocal of median germination time τ^−1^ (*h*^−1^) and maximum germination percentage *P*_*max*_ (%) of *Paecilomyces niveus* UBOCC-A-11024 **(A,F)**, *Mucor lanceolatus* UBOCC-A-109153 **(B,G)**, *Penicillium brevicompactum* UBOCC-A-110007 **(C,H)**, *Penicillium expansum* UBOCC-A-110032 **(D,I)** and *Penicillium roqueforti* UBOCC-A-113022 **(E,J)** on PDA medium (pH 4.2, 0.980 *a*_*w*_) at 25°C. Observed parameters (open circle), model fitted with Equation (4) (solid line).

The reciprocal of median germination times (τ^−1^) decreased when CO_2_ partial pressure increased from 0 to 70% (Figure 4). The model Equation (4) described this effect for the five tested species (Table 3) and provided three parameters, namely the reciprocal of median germination time under atmospheric partial pressure of CO_2_ ($\tau_{atm}^{-1}$), the CO_2_ partial pressure at which τ^−1^ is half of $\tau_{atm}^{-1}$ ([C*O*_2_]_50_) and the minimal inhibitory partial pressure of CO_2_ ([*CO*_2_]_*MI*_)_._ Regarding the $\tau_{atm}^{-1}$ parameter, it ranged between 0.0951 h^−1^ (namely 10.51 h) and 0.0535 h^−1^ (namely 18.69 h), depending on the fungal species. *M. lanceolatus* ($\tau_{atm}^{-1}$ = 0.0951 ± 0.0019 h^−1^) and *P. expansum* conidia ($\tau_{atm}^{-1}$ = 0.0719 ± 0.0013 h^−1^) germinated faster than those of the other tested species. Indeed, *P. brevicompactum, P. niveus* and *P. roqueforti* conidia had a median generation time > 16 h with $\tau_{atm}^{-1}$ of 0.0535 ± 0.0014 h^−1^, 0.0553 ± 0.0011 h^−1^ and 0.0599 ± 0.0015 h^−1^, respectively. Regarding the parameter [C*O*_2_]_50_, a CO_2_ partial pressure above 20% was necessary to double the median germination time. Indeed, [C*O*_2_]_50_ values were estimated at 20.54 ± 1.35% CO_2_, 53.65 ± 1.57% CO_2_ and 67.54 ± 2.74% CO_2_ for *P. brevicompactum, P. expansum* and *M. lanceolatus*, respectively. *P. roqueforti* [C*O*_2_]_50_ value (92.01 ± 12.90% CO_2_) was higher than the highest tested partial pressure (i.e., 70%). Regarding the parameter [*CO*_2_]_*MI*_, none of the tested CO_2_ partial pressure reduced τ^−1^ to 0 h^−1^. Indeed, [*CO*_2_]_*MI*_ estimated values were higher than 100% i.e., 102.79 ± 14.25%, 104.90 ± 8.14% and 124.36 ± 21.10% CO_2_ for *P. brevicompactum, P. expansum* and *M. lanceolatus*, respectively. For *P. roqueforti* and *P. niveus*, [*CO*_2_]_*MI*_ values higher than 10^4^ % CO_2_ were estimated.

**Table 3:** Secondary model parameters ± standard deviation obtained by fitting Equation (4) to reciprocal of median germination times τ^−1^ (h^−1^) or maximum germination percentages *P*_*max*_ (%) of *Paecilomyces niveus* UBOCC-A-11024, *Mucor lanceolatus* UBOCC-A-109153, *Penicillium brevicompactum* UBOCC-A-110007, *Penicillium expansum* UBOCC-A-110032 and *Penicillium roqueforti* UBOCC-A-113022 as a function of CO_2_ partial pressure in headspace.

**Fungal species**	**Parameters describing the effect of CO**_**2**_ **partial pressure on median germination time (**τ**)**					**Parameters describing the effect of CO**_**2**_ **partial pressure on maximal germination percentage (P**_*****max*****_**)**				
	**$\tau_{atm}^{-1}$*(h)***	**[*CO*_2_]_50_*(%)***	**[*CO*_2_]*_*MI*_ (%)***	***r*^2^**	**RMSE**	***P_*maxatm*_ (%)***	**[*CO*_2_]_50_*(%)***	**[*CO*_2_]*_*MI*_ (%)***	***r*^2^**	**RMSE**
*P. niveus*	0.0553 ± 0.0011	349.71 ± 734.57	>10^4^	0.70	0.002	–	–	–	–	–
*M. lanceolatus*	0.0951 ± 0.0019	67.54 ± 2.74	124.36 ± 21.10	0.95	0.004	–	–	–	–	–
*P. brevicompactum*	0.0535 ± 0.0014	20.54 ± 1.35	102.79 ± 14.25	0.98	0.002	98.6 ± 0.9	67.88 ± 0.35	68.24 ± 0.88	0.96	2.68
*P. expansum*	0.0719 ± 0.0013	53.65 ± 1.57	104.90 ± 8.14	0.98	0.002	–	–	–	–	–
*P. roqueforti*	0.0599 ± 0.0015	92.01 ± 12.90	>10^4^	0.92	0.003	98.8 ± 2.0	84.80 ± 9.98	92.18 ± 15.71	0.69	4.89

*The parameters $\tau_{atm}^{-\text{1}}$ (h^−1^) and P_maxatm_ are the respective values of τ^−1^ and P_max_ in atmosphere. The parameter [CO_2_]_50_ (%) is the CO_2_ partial pressure at which τ^−1^ half of $\tau_{\text{atm}}^{-\text{1}}$ or P_max_ is half of P_maxatm_. The parameter [CO_2_]_MI_ (%) is the CO_2_ partial pressure at which τ^−1^ or P_max_ = 0. The accuracy of the model was evaluated using the root mean square error (RMSE) and the determination coefficient r^2^. (–) Model not adjusted*.

#### Modeling the effect of carbon dioxide partial pressure on maximum percentage of germination P_max_

The effect of O_2_ partial pressure on the maximum percentage of germination (*P*_*max*_) varied according to the studied fungal species (Figures 4F–J). As already observed for O_2_, *P*_*max*_ of *P. niveus, M. lanceolatus* and *P. expansum* were not affected by any of the tested CO_2_ levels and had a constant value close to 100% (Figures 4F,G,I). Therefore, the CO_2_ effect on *P*_*max*_ could not be modeled. For *P. brevicompactum* and *P. roqueforti, P*_*max*_ parameter remained constant at CO_2_ levels below 50 and 40% respectively followed by a decrease at the highest CO_2_ partial pressures (Figures 4H,J). Using our model Equation (4), we could estimate *P*_*maxatm*_ (maximum germination percentage under atmospheric CO_2_ partial pressure), [C*O*_2_]_50_ (CO_2_ partial pressure at which *P*_*max*_ is half of *P*_*maxatm*_) and [*CO*_2_]_*MI*_ (minimal inhibitory CO_2_ partial pressure) for the latter species as shown in Table 3. Regarding *P*_*maxatm*_, more than 98% of *P. brevicompactum* and *P. roqueforti* conidia germinated under atmospheric CO_2_ partial pressure (Table 3). Regarding both [C*O*_2_]_50_ and [*CO*_2_]_*MI*_ parameters, the inhibition of 50 % or 100 % of the inoculated conidia required an increase in CO_2_ level up to 67.88 ± 0.35% CO_2_ and 84.80 ± 9.98% CO_2_ and, 68.24 ± 0.88% CO_2_ and 92.18 ± 15.71% CO_2_ for *P. brevicompactum* and *P. roqueforti*, respectively. It is worth mentioning that these values were not within the CO_2_ levels evaluated in the present study.

## Discussion

The present work aimed at modeling the respective effects of O_2_ and CO_2_ partial pressure on the conidial germination kinetic of five fungal species encountered in dairy foods. To the best of our knowledge, this the first time that conidial germination under modified atmosphere is assessed on solid medium using phase-contrast microscopy. Indeed, previous studies were performed in liquid media (Nguyen Van Long and Dantigny, 2016). Even though PDA cannot be considered as a food matrix, it is more representative of solid foods which are usually contaminated on their surfaces by conidia which are exposed to the gas headspace of the package. The method and experimental device developed in the present study can be considered as representative of MAP as well as CAS because gas composition was set up before package sealing and remained constant throughout germination kinetic assessment with O_2_ and CO_2_ partial pressure down to 0.36 ± 0.05% and up to 69.00 ± 1.76%, respectively. In addition, the fact that each plate was packed individually allowed gas composition measurements throughout the experiment without modifying the headspace gas composition of other incubated Petri dishes. Indeed, a static gas composition must be maintained to accurately model O_2_ and CO_2_ effects using Gamma-type models.

However, in real foods under MAP, the gas composition can vary during storage and depends on gas transfer between the food itself, the packaging headspace and the storage environment (Chaix et al., 2015). Indeed, gas transfer between the food and the packaging headspace depends on abiotic (gas solubilization, diffusion, and subsequent chemical reactions) and biotic factors (microbial respiration/fermentation and food respiration in the case of respiring products) while transfer between the packaging headspace and the storage environment mainly depends on the gas permeability of the packaging material (Chaix et al., 2015). In future work, such transfer could also be taken into account with different culture devices based on the present method, i.e., compartmentalized bag made from high oxygen barrier plastic film.

To our best knowledge, the models describing the effects of O_2_ and CO_2_ partial pressure on conidial germination parameters (Equations 2–4) were applied for the first time in the present work. Despite an accurate fitting of the models to the observed data (Figures 3, 4), the *r*^2^ and RMSE values were not as good as expected in certain cases (Tables 2, 3). For example, when modeling *P. roqueforti* germination as a function of the O_2_ partial pressure, *r*^2^ values of 0.45 and 0.68 were obtained for the models describing τ and *P*_*max*_ parameters, respectively. These values were mainly due to the asymptotic shape of the curve, especially for data obtained at O_2_ partial pressure below 1%. However, the models presented in our study were still relevant as they could be used as part of a gamma-concept-based approach to take into account O_2_ and/or CO_2_ effects as multiplicative factors (Zwietering et al., 1993). In this perspective, it would be of interest to investigate their interaction with other environmental factors, as previously shown for CO_2_ and *a*_*w*_ (Samapundo et al., 2007). In addition, it is worth mentioning that these models (Equations 2–4) provide biologically meaningful parameters (median germination time τ and maximum germination percentage *P*_*max*_) which can then be compared and biologically interpreted. For example, an increase of the τ value (as described by Equation (2) will reflect a delay in conidial germination (fungistatic effect) while a decrease of the *P*_*max*_ value (as described by Equation (3) will reflect an inhibition of the germination of certain conidia within a population. Concerning the *d* parameter, predictions of conidial germination will be facilitated by the use of constant *d* values, namely the mean values estimated for each fungal species. Indeed, no significant correlation was observed between *d* and the headspace gaseous composition in the present study.

As other strictly aerobic microorganisms, food spoilage molds have an absolute requirement of O_2_ to produce adenosine tri-phosphate (ATP) *via* the oxidative phosphorylation pathway (Bailey-Serres and Chang, 2005), although a wide diversity of molds are able to grow under reduced O_2_ partial pressure as low as 1% (Nguyen Van Long and Dantigny, 2016). As expected, none of the five tested fungi were able to germinate under strict anaerobic conditions despite a high tolerance to O_2_ reduction (down to 0.36%) was observed for certain fungal species. The highest tolerance was exhibited by *M. lanceolatus* and *P. roqueforti* with [O_2_]_50_ values lower than the lowest tested O_2_ partial pressure. Consistent with these results, the growth of *Mucor ambigus* on Koji agar and *P. roqueforti* on wheat extract agar was reported at O_2_ partial pressure of 0.10% (Yanai et al., 1980) and 0.14% (Magan and Lacey, 1984), respectively. Previous studies also reported growth of *Byssochlamys nivea* (teleomorph of *P. niveus*) on cheddar cheese (Taniwaki et al., 2001), *P. brevicompactum* on Czapek agar (Yang and Lucas, 1970) and *P. expansum* on apple puree agar medium (Baert et al., 2007) at O_2_ partial pressure as low as 1%, which is a higher value than that observed in the present work.

Interestingly, all the aforementioned publications studied radial growth as a biological response even though spores were used as an inoculum. In this case, it could be ambiguous to determine precisely whether O_2_ reduction affected successively conidial germination and radial growth or only one specific developmental stage. As the O_2_ limits described in the present study are in the same order of magnitude as previously reported for radial growth, it can be hypothesized that delays in radial growth caused by low O_2_ partial pressure principally result from significant delays in the germination process. To test whether hyphal elongation is specifically affected by oxygen reduction, further experiments should be performed with conidia pre-incubated under atmospheric condition (until full germination) followed by incubation under modified-atmosphere. In addition, a better understanding of the high tolerance to low O_2_ level displayed by filamentous fungi will require further investigation of their oxygen sensing mechanisms. Indeed, direct and indirect oxygen reduction sensors were extensively studied in plant cells (Bailey-Serres and Chang, 2005; Bailey-Serres et al., 2012), animal cells (Bruick, 2003) and yeasts (Kwast et al., 1998; Poyton, 1999) but not in filamentous fungi.

In the literature, it is generally reported that fungal growth is reduced but rarely inhibited at CO_2_ partial pressure ranging from 50 to 90% (Nguyen Van Long and Dantigny, 2016). This is consistent with our results as the five tested fungi were able to germinate at 70% CO_2_ despite a delay and/or a reduction of the maximum germination percentage was observed depending on the fungal species. It is also worth mentioning that [C*O*_2_]_50_ and [CO_2_]_MI_ values higher than 100% were estimated for *P. niveus* and *P. roqueforti*. Such hyperbaric CO_2_ pressure cannot be obtained with our modified-atmosphere culture device. Therefore, these values should be taken with caution as they are model projections (Equations 2–4) and thus, could not be validated. Nonetheless, the highest CO_2_ tolerance was exhibited by *P. niveus* and *P. roqueforti* with the highest [CO_2_]_50_ (estimated CO_2_ level to double the median germination time) and [CO_2_]_MI_ (estimated CO_2_ level to prevent conidial germination) values. Consistent with these results, growth of *B. nivea* (teleomorph of *P. niveus*) on PDA and *P. roqueforti* on Czapek yeast extract agar was reported at CO_2_ partial pressure up to 80% (with O_2_ partial pressure set at 20%) (Taniwaki et al., 2010) and 99% (O_2_ partial pressure 1%) (Nielsen and Rios, 2000), respectively but was not tested at higher partial pressures. Previous studies also reported growth of *M. plumbeus* on PDA (Taniwaki et al., 2010), *P. brevicompactum* on Czapek agar (Yang and Lucas, 1970) and *P. expansum* on apple (Moodley et al., 2002) at CO_2_ partial pressure ranging between 80 and 95% (in presence of residual O_2_), which are higher values than the maximum CO_2_ level tested in the present study. Taken together, the present results confirmed that in the presence of 5% O_2_, a CO_2_ level up to 70% was not sufficient to prevent conidial germination of the tested fungi. As discussed above for O_2_, it can be hypothesized that delays in radial growth reported in the aforementioned studies also mainly resulted from delayed germination. Interestingly, ([CO_2_]_50_) or ([CO_2_]_MI_) were often estimated above 100%, suggesting that pressurized CO_2_ would be required to obtain the corresponding effects. High-pressure carbon dioxide (HPCD) is currently used as an alternative to thermal inactivation of microorganisms for increasing the shelf life of heat-sensitive foods (Garcia-Gonzalez et al., 2007). In the case of HPCD, the bactericidal effect of CO_2_ was extensively studied and identified as resulting from a combination of interactions between dissolved CO_2_ and the plasma membrane, inhibition of enzymatic reactions through internal pH modifications and carboxylation/decarboxylation reactions, and modifications of osmotic regulation through intracellular electrolyte precipitation (Garcia-Gonzalez et al., 2007). Similar mechanisms may be involved at atmospheric pressure (e.g., in MAP and CAS). In the case of conidia, it would be interesting to investigate whether the cell wall composition (Sewall et al., 1990; Latgé and Beauvais, 2014) or the high intracellular content of compatible solutes (Wyatt et al., 2013) contribute to the important tolerance to increased CO_2_ partial pressure as observed in the present work.

Interestingly, for *P. brevicompactum* and *P. roqueforti*, we observed that both *P*_*max*_ and τ parameters were simultaneously affected by O_2_ reduction or CO_2_ increase. Indeed, half of the inoculated *P. brevicompactum* and *P. roqueforti* conidia were still able to germinate at 0.25% and 0.32% O_2_, respectively, with median germination times 6 and 3.5-fold longer than under atmospheric conditions. Similarly, half of the inoculated *P. brevicompactum* and *P. roqueforti* conidia remained able to germinate at 67.88% and 84.80% CO_2_, respectively, with median germination times 8.8 or 1.9-fold longer than under atmospheric conditions. This result shows that a sub-population among the conidial population is less susceptible to O_2_ reduction or CO_2_ increase despite their germination time is longer. Consequently, it can be hypothesized that tolerance to MAP could be related to heterogeneity among conidia population. A similar hypothesis was previously made to explain the high tolerance of *Zygosaccharomyces bailii* to weak-acid preservatives (Stratford et al., 2014). Overall, these results suggest that accurate prediction of conidial germination in foods could be impaired given that such an heterogeneity exists within conidial population and that mold spoilage generally arises from a single conidia (Burgain et al., 2013).

As a conclusion, the present study confirmed that a modified atmosphere, consisting of O_2_ or CO_2_ partial pressure higher than 1 and 70%, respectively, was not sufficient to prevent conidial germination of the five studied fungal species, all of which are frequently encountered as food spoilers or, in the case of *M. lanceolatus* and *P. roqueforti*, utilized as cheese ripening cultures. As part of the hurdle technology, O_2_ and CO_2_ hurdle effects should be combined together as well as with other biotic and abiotic factors to prevent fungal spoilage. The effect of combined factors on mold development can then be predicted with gamma-type models assuming multiplicative effects of the different hurdles. In this perspective, the developed culture device and predictive models of this study could be of interest for food mycologist and industrials to describe O_2_ and CO_2_ level effects on conidial germination.

## Author contributions

NN, VV, and KR designed the experiments and prepared the manuscript. NN and MB performed the experiments. OC, LC, and NN computed the modeling. JM led the research.

### Conflict of interest statement

The authors declare that the research was conducted in the absence of any commercial or financial relationships that could be construed as a potential conflict of interest.
